# Losartan Treatment Protects Retinal Ganglion Cells and Alters Scleral Remodeling in Experimental Glaucoma

**DOI:** 10.1371/journal.pone.0141137

**Published:** 2015-10-27

**Authors:** Harry A. Quigley, Ian F. Pitha, Derek S. Welsbie, Cathy Nguyen, Matthew R. Steinhart, Thao D. Nguyen, Mary Ellen Pease, Ericka N. Oglesby, Cynthia A. Berlinicke, Katherine L. Mitchell, Jessica Kim, Joan J. Jefferys, Elizabeth C. Kimball

**Affiliations:** 1 The Glaucoma Center of Excellence, Wilmer Ophthalmological Institute, Johns Hopkins University, Baltimore, Maryland, United States of America; 2 Department of Mechanical Engineering, Johns Hopkins University, Baltimore, Maryland, United States of America; Instituto Murciano de Investigación Biosanitaria-Virgen de la Arrixaca, SPAIN

## Abstract

**Purpose:**

To determine if oral losartan treatment decreases the retinal ganglion cell (RGC) death caused by experimental intraocular pressure (IOP) elevation in mice.

**Methods:**

We produced IOP increase in CD1 mice and performed unilateral optic nerve crush. Mice received oral losartan, spironolactone, enalapril, or no drug to test effects of inhibiting angiotensin receptors. IOP was monitored by Tonolab, and blood pressure was monitored by tail cuff device. RGC loss was measured in masked axon counts and RGC bodies by β-tubulin labeling. Scleral changes that could modulate RGC injury were measured including axial length, scleral thickness, and retinal layer thicknesses, pressure-strain behavior in inflation testing, and study of angiotensin receptors and pathways by reverse transcription polymerase chain reaction, Western blot, and immunohistochemistry.

**Results:**

Losartan treatment prevented significant RGC loss (median loss = 2.5%, p = 0.13), while median loss with water, spironolactone, and enalapril treatments were 26%, 28% and 43%; p < 0.0001). The lower RGC loss with losartan was significantly less than the loss with spironolactone or enalapril (regression model p = 0.001; drug treatment group term p = 0.01). Both losartan and enalapril significantly lowered blood pressure (p< 0.001), but losartan was protective, while enalapril led to worse than water-treated RGC loss. RGC loss after crush injury was unaffected by losartan treatment (difference from control p = 0.9). Survival of RGC in cell culture was not prolonged by sartan treatment. Axonal transport blockade after 3 day IOP elevations was less in losartan-treated than in control glaucoma eyes (p = 0.007). Losartan inhibited effects of glaucoma, including reduction in extracellular signal-related kinase activity and modification of glaucoma-related changes in scleral thickness and creep under controlled IOP.

**Conclusions:**

The neuroprotective effect of losartan in mouse glaucoma is associated with adaptive changes in the sclera expressed at the optic nerve head.

## Introduction

Glaucoma is the most common preventable cause of blindness worldwide [1], and its damaging effects are known to be mediated through alterations at the optic nerve head (ONH) produced by the action intraocular pressure (IOP) [2,3]. Engineering models of ocular tissues that describe IOP-induced effects show that peripapillary sclera (PPS) behavior is important in determining the effect of IOP on the ONH [4,5,6]. Risk factors for human glaucoma include features related to scleral anatomy or physiology, including axial myopia, corneal hysteresis, and corneal thickness [7]. In human glaucoma eyes the sclera is stiffer by *in vivo* indirect measurement [8] and by *in vitro* inflation testing [9], and undergoes alterations in PPS collagen fiber orientation [10].

The sclera is substantially altered in experimental glaucoma in mice [11] including axial elongation, increase in stiffness on inflation testing [12], loss of non-fibrillar matrix [13] thickening and reorientation of collagenous fiber layers, decreased scleral permeability [14], increased scleral fibroblast activity and division, and increase in integrin-linked and actin-cytoskeletal signaling by proteomic analysis [15]. Alterations in PPS affect susceptibility to experimental glaucoma damage. Mice with a mutated connective tissue gene for collagen 8α2 have larger eyes, with stiffer sclera at baseline and they resist glaucoma injury more than do wild type C57BL/6 mice [16]. Increased scleral cross-linking by application of glyceraldehyde led to increased retinal ganglion cell (RGC) death in experimental mouse glaucoma [17]. It is therefore likely that both the baseline state of the sclera and its dynamic alteration could affect the manner in which IOP is translated into a damaging stimulus in glaucoma.

The transforming growth factor β (TGFβ) pathways are activated in experimental and human glaucoma in trabecular meshwork [18] and ONH [19,20]. Proteomic analysis of sclera in mice with experimental glaucoma show >2-fold increases in thrombospondins 1 and 4, known activators of TGFβ [15]. The selective angiotensin 1 receptor (AT1R) inhibitor, losartan, suppresses Smad2 phosphorylation and TGFβ expression in its canonical pathway, as well as inhibiting phosphorylation of extracellular signal-related (ERK) in a parallel pathway. Blockade of AT1Rs increases AT2R activation, reducing TGFβ stimulation [21]. Overactivity of TGFβ in the Marfan syndrome leads to aortic dissection [22,23], and selective AT1R inhibition by losartan is beneficial in mouse Marfan models and has entered clinical trials [24]. Candesartan, an AT1R inhibitor, was reported to decrease RGC loss in experimental rat glaucoma with oral dosing [25], no detailed studies were performed in that report to show the mechanism by which the drug acted.

We hypothesize that losartan-induced inhibition of TGFβ signaling in a mouse model of glaucoma would alter RGC survival by modifying the scleral response to chronically elevated IOP. Potential therapeutic alterations of the sclera could supplement IOP-lowering therapy in human glaucoma by reducing IOP-generated stress at the ONH [26].

## Methods

### Experimental Mice

We used 497 CD1 albino, female mice that were 2 months of age at the start of each experiment (Table 1; Charles River, Inc., Wilmington, MA). Animals were treated in accordance with the ARVO Statement for the Use of Animals in Ophthalmic and Vision Research and at no time were blinding conditions induced in both eyes of the same animal. All protocols were approved and monitored by the Johns Hopkins University School of Medicine Animal Care and Use Committee. The numbers of animals tested in each portion of the experiments are given below.

**Table 1:** Experimental study groups and the number of animals within each.

Study	Number of Animals
IOP Measurement/ Bead Injection Glaucoma	200
Sclera Thickness Measurement	120
Biomechanical Inflation Testing	59
Immunoblot	40
Blood Pressure Measurements	25
Optic Nerve Crush	22
Axonal Transport Blockade-APP	15
Immunostaining	12
qRT-PCR	4
**Overall**	**497**

### Experimental Drug Treatment

Animals were treated with losartan (Cozaar, Merck, Whitehouse Station, NJ), spironolactone (Aldactone, Pfizer, New York, NY), or enalapril (Santa Cruz, Inc., Dallas, TX) in their drinking water starting 2 weeks prior to experimental induction of elevated IOP or other procedures. A fourth group of mice received only drinking water without added drug. Treatment was continued until cessation of the experiment. Losartan was dissolved in water and filtered to a final concentration of 0.6g/L, providing an estimated daily dose of 40-60mg/kg/day [27]. Spironolactone was dissolved first in a small amount of ethanol and then diluted with water to a final ethanol concentration of approximately 0.1% [28,29] and delivered at a dose of approximately 20 mg/kg/day. During the experiment, it was detected that the spironolactone had not remained in solution in the drinking water bottles, and we therefore concluded that this group did not constitute a control for lowered blood pressure to the losartan group. However, it was retained as a second glaucoma group to compare with losartan and water groups. Enalapril dosing at 0.15 g/L gave an estimated daily dose of 10–15 mg/kg/day. This provided a second blood pressure lowering control to compare with losartan, as well as offering a means to differentiate between AT1R inhibition alone (losartan) and both AT1R and AT2R inhibition (enalapril is an ACE inhibitor).

### IOP measurement

For IOP measurements with the Tonolab tonometer (TioLat, Inc., Helsinki, Finland), mice were anesthetized by inhalation of a mixture of oxygen and isoflurane using the RC2- Rodent Circuit Controller (VetEquip, Inc., Pleasanton, CA). We have published the accuracy of the Tonolab in normal and glaucoma mice [30,31], but to confirm that losartan treatment did not affect the accuracy of IOP measurement, we determined that the calibration of the Tonolab was accurate by comparing its readings with IOP set by cannulation and attachment to a reservoir whose height determined IOP in 30 mice. This was performed in masked fashion in mice treated with losartan in which one eye had undergone IOP elevation by bead injection for either 1 week or 6 weeks. Readings were taken stepwise at reservoir heights corresponding to pressures of 10, 20, 30, 40, and 50 mm Hg. We collected 5 means of 6 measurements at each set pressure setting (a total of 30 measurements per eye) and used the median of the 5 means as final IOP reading.

As in prior reports, we calculated the IOP exposure over time in each animal by integrating the cumulative IOP increase in bead-injected (experimental glaucoma) eyes to that animal’s fellow uninjected eye. This was done by calculating the area under the IOP over time curve for injected eye and subtracting the area under the curve of the control eye for all periods during the 6 weeks of glaucoma experiments between injection and sacrifice. This value is called the positive integral IOP with units of mm Hg/days. In regression models, we also estimated the IOP exposure among groups of mice by comparing the peak IOP and the average difference between elevated and fellow normal eye during the 6 week experiment.

### Blood pressure measurement

Blood pressure was measured in 25 awake, unanesthetized mice with a Visitech BP-2000 Series II (Visitech System, Apex, NC) which measures blood pressure and pulse by means of an inflatable tail cuff and a photoplethysmographic LED system. The mouse tail was placed through a tail cuff and held stationary by adhesive tape in a holder between a light source and a photoresistor, with sampling 200 times per second [32]. Mice were placed on a plate heated to 38°C to avoid vasoconstriction and trained on the system 4 times in one week prior to collecting measurements [33]. Animals were treated with spironolactone, losartan, enalapril, or standard water alone for two weeks prior to blood pressure measurement. Each blood pressure measurement was calculated as the mean of 20 cuff inflation-deflation trials, with measurements on at least 3 separate days, calculating an overall mean blood pressure for each mouse.

### Bead Injection Glaucoma Model

In the glaucoma experiment, 200 mice were anesthetized with a mixture of ketamine (Fort Dodge Animal Health, Fort Dodge, IA), xylazine (VedCo Inc., Saint Joseph, MO), and acepromazine (Phoenix Pharmaceuticals, Burlingame, CA) at 50, 10 and 2 mg/kg, respectively. The dosage and time of anesthesia was controlled and has been standardized as previously published [11]. Then, one anterior chamber was injected with Polybead Microspheres^®^ (Polysciences, Inc., Warrington, PA, USA), using the 4+1 protocol [11], consisting of 2 μl of 6 μm diameter beads, then 2 μl of 1 μm diameter beads, followed by 1 μl of viscoelastic compound (10 mg/ml sodium hyaluronate, Healon; Advanced Medical Optics Inc., Santa Ana, CA). The injections were made through a glass cannula with 50 μm tip diameter, connected to a Hamilton syringe (Hamilton, Inc., Reno, NV). IOP was measured immediately after injection, and at 3 days, 1 week, 2 weeks and 6 weeks, using the Tonolab tonometer. Investigators were masked as to treatment group during IOP measurements. The experimental and control animals were treated in masked fashion on the same days, interchangeably, so that any difference in the state or effect of anesthesia would be random and would not lead to any systematic bias. At all steps of the experiment, the solutions and tissues were coded to mask the participants.

### Biomechanical Inflation Testing

Eyes of 59 CD1 mice, 4–6 months old, were inflation tested post-mortem in procedures previously published [12]. Inflation testing was carried out on 5 groups, eyes that had undergone 6 weeks of elevated IOP with either losartan treatment (N = 20) or drinking water (N = 19), their fellow control eyes (N = 19 water only, N = 20 losartan only), and animals that had undergone exposure to drug alone without glaucoma in either eye (N = 20). Animals were euthanized with a mixture of ketamine, xylazine, and acepromazine as described above, the superior cornea was marked for orientation, the eyes were enucleated and placed in 0.1 M phosphate buffer. The axial length was measured from the cornea to the temporal optic nerve margin, while width was measured at the largest dimension at the equator, midway between cornea and optic nerve, in both the nasal-temporal axis and the superior—inferior axis with a digital caliper (Instant Read Out Digital Caliper, Electron Microscopy Sciences, Hatfield, PA, USA). The anterior portion of the whole, enucleated eye was glued into a custom fixture with the anterior chamber cannulated by a 30 gauge needle. Then, the eye was placed in a water bath at 20°C under a dissecting microscope (Carl Zeiss Microimaging, Thornwood, NY). The globe was oriented with the superior pole toward the camera, providing a view of the nasal-temporal sclera in the measuring plane. For scleral inflation, IOP was controlled through the cannula and measured with an in-line pressure transducer (Sensotech Inc, Wayne, NJ). Scleral displacement was measured during inflation using a two-dimensional digital image correlation (DIC) system, which consisted of a CCD video camera (Grasshopper, model Gras-20S4M-C, Point Grey Research, Inc., Richmond, BC, Canada) attached to the dissecting microscope and a commercial DIC software package (Vic-2D, Correlated Solutions, Columbia, SC); the camera captured images every 2 seconds during the inflation protocol. The eyes underwent a load—unload inflation from a reference pressure of 6–10 mm Hg to 30 mm Hg and back to reference pressure, with a loading rate of 0.25 mm Hg/s. Then, the pressure was raised to 30 mm Hg and held for 30 minutes with displacement/strain data extracted every 5 minutes to estimate creep rate. The strains in two directions, circumferential and meridional, were calculated for each eye from the DIC-determined position and displacement measurements as previously described [12]. Unsuccessful inflation tests consisting of; obvious leakage from cannulation, eyes that detached from fixture, technical failure or did not complete the protocol were not included in the analysis.

### Scleral Thickness Measurements

In 60 mice, scleral thickness was measured on both fresh, unfixed sclera under a dissecting microscope and on histological sections embedded in epoxy, as described in a previous publication [13]. For fresh tissue thickness measurements, an eyepiece micrometer within the Zeiss dissecting microscope (Electron Microscopy Sciences. Hatfield, PA) was used to measure scleral specimens prepared with a razor blade to a resolution of ± 0.01 mm. Three strips, each 0.33 mm wide and 2.5 mm long, were cut from the superior sclera. Thickness measurements were made at 6 locations, every 0.5 mm, starting at the ONH and continuing anteriorly to the limbus.

For comparison to fresh measurements, fixed tissues were prepared in the following manner. Sixty animals were euthanized and then perfused with 4% paraformaldehyde in 0.1 M phosphate buffer. The same eyes were also used for histological retinal analysis and optic nerve axon counts. After perfusion, eyes were enucleated and superior sclera was marked and the optic nerve was removed. The ONH was removed using a trephine and fixed along with the remaining sclera in 1% osmium tetroxide, dehydrated in ethanol, and stained with 1% uranyl acetate for 1 hour. Sclera was embedded in epoxy resin and 1 micron sections were stained with 1% toluidine blue. Images were taken using a 100x oil objective on a Zeiss light microscope (Carl Zeiss MicroImaging, Thornwood, NY) and a total of nine measurements were made for each animal and analyzed with Metamorph Image Analysis software (Molecular Devices, Downington, PA).

### Optic Nerve Crush Experiment

CD1 mice were pretreated for two weeks with losartan (13 mice) or a vehicle control (9 mice), then subjected to a unilateral optic nerve crush with fine forceps for 9 seconds, with the fellow eye undergoing sham surgery omitting the actual crush injury. For 13 more days, the animals were either treated with losartan or vehicle, then eyes were harvested and stained with a mouse monoclonal against NeuN (Cat # MAB377, EMD Millipore, Billerica MA), at 1:500 concentration, and rabbit poly clonal for gamma-synuclein (Sncg; kindly provided by N. Marsh-Armstrong), 1:500 concentration. Secondary antibody used for NeuN was donkey anti mouse AlexaFluor^®^ 568 (Cat #A10037 Invitrogen, Carlsbad, CA) at 1:1000 concentration and for gamma-synuclein was AlexaFluor^®^ 488 labeled goat anti rabbit (Cat #A11008 Invitrogen, Carlsbad, CA) was used at 1:1000 concentration. An observer masked to treatment group acquired 8 field images per retina, using a 20x lens on a Zeiss Confocal LSM 510 (Zeiss MicroImaging LLC, Thornwood, NY). Retina images were analyzed with a custom algorithm created using the Cellomics Target Activation (Thermo Fisher Scientific, Pittsburgh, PA) image-analysis software package. With this algorithm, we identified Scng stained cells based on their intensity, then quantitated Scng stained cells that also had NeuN staining above an intensity threshold.

### Histological Observations

For histological observations and for measurement of the outcome of glaucoma experiments to assess RGC axon loss, perfusion-fixed globes and optic nerves (N = 81 and N = 50, respectively, this tissue was acquired from animals in bead glaucoma study listed above) were post-fixed in 1% osmium tetroxide, epoxy-embedded, and sectioned at 1 μm thickness. During the entire process of axon counting, the observers were masked to the treatment group. Digital images of optic nerve cross-sections were taken at low power to measure the total optic nerve area. Then, five, 40 x 40 μm fields (100x magnification, Cool Snap camera, Metamorph Image Analysis software; Molecular Devices, Downington, PA) were acquired, representing a random 9% sample of the total nerve area. A masked observer edited non-axonal elements from each image and the program calculated axon density. Average axon density/mm^2^ was multiplied by the individual nerve area to estimate axon number. Experimental eyes were compared to the mean axon number in pooled, fellow eye nerves of the same experimental group to yield percent axon loss.

In addition, from 1 μm epoxy sections of retina, we quantitatively measured retinal layer thickness in losartan-treated and in control eyes to evaluate possible effects of treatment other that in the RGC layer (N = 20). Thicknesses of the inner nuclear and outer nuclear layers were measured at 6 locations on each section by a masked observer.

For counts of numbers of fibroblasts and percentage of dividing fibroblasts in sclera and counts of RGC bodies in retina, whole mounted scleras and retinas were used. Twenty mice were sacrificed by exsanguination under general intraperitoneal anesthesia and perfused transcardially with 4% paraformaldehyde in 0.1 M phosphate buffer solution (PB), pH 7.2. The superior pole of each eye was marked for orientation of specimens, and the eyes were enucleated. After a series of washes in PB and PB containing 0.3% Triton (PBT), scleras were segmented into quadrants representing superior, nasal, inferior and temporal positions and retinas were incised to lie flat.

For exposure to primary antibody, 20 sclera samples (5 water only, 5 water + glaucoma, 5 losartan only, and 5 losartan + glaucoma) were incubated overnight at 4°C with polyclonal rabbit anti-Ki67 raised against human Ki67 peptide (Cat# ab15580, Abcam, Cambridge, MA). The antibody was diluted 1:100 in phosphate buffered saline with 0.5% Triton (PBT) with 10% normal goat serum (NGS). After incubation in primary antibody solution, samples were washed in PBT and incubated in goat anti-rabbit AlexaFluor^®^488 (Cat # A11008, LifeTechnologies, Grand Island, NY) secondary at 1:500 for 2 hours at room temperature. After final antibody incubation, nuclei were stained with DAPI at 5ug/ul (Invitrogen, Grand Island, NY). Scleras were coverslipped with DAKO mounting media (DAKO, Carpenteria, CA).

The number of DAPI-labeled nuclei in each scleral quadrant was quantified using Stereo Investigator software (MicroBrightField, Williston VT) integrated with an ECLIPSE E600 microscope (Nikon, Japan) with a 3-Chip CCD color video camera (HV-C20, Hitachi, Japan) and motorized stage and microcator attachment (Heidenhain EXE 610C, Schaumburg, IL). All DAPI-labeled cells with the elongated shape and ovoid nucleus typical for fibroblasts were counted from a zone approximately 10 μM thick in the outer sclera. The density of cells per unit area of DAPI stained cells and number and percent labeling positively for Ki67 were analyzed by scleral region (from close to the peripapillary area to the equator) and by experimental glaucoma compared to control.

Retinal whole mounts (2 water only, 2 water + glaucoma, 2 losartan only, 2 losartan + glaucoma) fixed with 4% paraformaldehyde were incubated overnight in monoclonal mouse anti-β-tubulin raised against rat brain microtubules (Catalog# MMS-435P, Covance, Inc., Princeton, NJ) at 1:500 dilution. AlexaFluor^®^ 488 labeled goat anti mouse secondary antibody (Cat #A11001 Invitrogen, Carlsbad, CA) was used to detect the primary antibody. Nuclei were stained with DAPI during the final wash. Samples were imaged with a Zeiss LSM 510 Meta Confocal Microscope (Zeiss MicroImaging LLC, Thornwood, NY). Four 40× images were taken in each of 4 retinal quadrants. Manual quantification of RGC identified as positive for β-tubulin was performed in masked fashion from images, using Metamorph Image Analysis software (Molecular Devices, Downington, PA, USA).

### Quantification by reverse transcription—polymerase chain reaction (RTPCR)

We quantified gene expression of AT1aR, AT1bR, and AT2R receptors in four normal CD1 mouse eyes. The following organs were harvested and grouped by organ: adrenal gland (4 samples), kidney (1/3 sample), retina (4 samples), sclera (4 samples), and optic nerve (4 samples). Tissues were homogenized, and RNA was isolated by RNeasy Mini Plus Kit (Qiagen, Valencia, CA). Synthesis of complementary (cDNA) was completed using 1 μg of total RNA using a Synthesis Kit for qRT-PCR (Bio-Rad, Hercules, CA). RT-PCR was performed on a CFX384- Real Time System Machine (Bio Rad), with each reaction consisting of 20 μL containing 10 μL SsoAdvanced Universal SYBR Green Supermix (Bio Rad), cDNA, and 0.6 μL AT receptor primer.

To amplify the receptors the following sequence code was used for AT1aR primer [34] FWD- 5’- GCTGGCAGGCACAGTTACAT- 3’, and REV- 5’- GACACTGGCCACAGTCTTCA- 3’. The AT1bR primer sequence code used was FWD- 5’- TTTTCCCCAGAGCAAAGCTA- 3’, and REV- 5’- CCCTCCCCCAAATCAATAGT- 3’ (Integrated DNA Technologies, Coralville, IA). The AT2R primer [35] used was FWD- 5’- GTCAAGGAAAAGGGTATACTCCAAGG-3’, and REV- 5’- GTGTGCTCAGGCTCACCATTG- 3’. Glyceraldehyde 3-phosphate dehydrogenase (GAPDH) primer was used as reference gene, forward primer 5’- TGAAGGTCGGAGTCAACGGATTTGGT-3’ and reverse primer 5’-CATGTGGGCCATGAGGTCCACCAC-3’. The thermocycling program consisted of: denaturing at 95°C for 30s, 95°C for 20s, followed by 45 annealing cycles at 60°C for 15s for AT1aR, and AT1bR primers. The AT2R primers required an annealing temperature of 65°C, while an annealing temperature of 55°C for GAPDH was used. Cycling threshold or crossing point (Cq) value for each sample was based on the cycle at which fluorescence from amplification exceeded the background fluorescence. Relative expression values were calculated by dividing the GAPDH primer treated tissue quantification cycle (Cq) value by the mean Cq value for AT1A, AT1B, or AT2 primer tissue. A lower value than 1 indicates less of that particular receptor in tissue compared to GAPDH.

### Western blot methods

Western blot quantification of proteins was performed with retina, sclera and optic nerve from control, losartan treated, and glaucoma eyes with and without losartan treatment. Samples of each tissue type from 10 animals per treatment group (total 40) were collected, with glaucoma eyes studied after IOP elevations for 3 days and 1 week. Briefly, eyes were excised, tissues separated and frozen a slurry of dry ice/ethanol in microcentrifuge tubes. Proteins were extracted using the Total Protein Extraction Kit (EMD Millipore, Inc., Billerica, MA). Tissues were placed in extraction solution and macerated with a motorized pestle (Kimble-Kontes, Hainesport, NJ), taken through freeze thaw cycles, and centrifuged to remove undissolved material. Sample protein concentrations were determined using EZQ Protein Quantification Kit (Life Technologies, Grand Island NY). Equal protein amounts for each sample were run on 4–10% Bis-Tris gels (Novex, Thermo Fisher Scientific, Waltham MA) and then wet transferred to polyvinylidene difluoride membranes (Bio-Rad, Hercules, CA). Membranes were washed in PBS containing 1% Tween-20 (PBT) (Sigma, St. Louis, MO). PBT containing 5% non-fat dry milk was used as blocking reagent and blots were probed with the following antibodies diluted in PBT/5% non-fat milk: rabbit monoclonal against synthetic phosphopeptide to human Smad3 at 1:700 (Cat #ab52903, Abcam, Cambridge, MA, this and other antibodies along with their company and dilution information can be found in Table 2); rabbit monoclonal synthetic phosphopeptide to human Smad2 at 1:500 (Cat#3108, Cell Signaling, Danvers, MA); a rabbit monoclonal to synthetic human Smad2/3 at 1:1000 (Cat #8685, Cell Signaling, Danvers, MA); rabbit monoclonal to synthetic phosphopeptide of human pErk1/2 at 1:4000 (Cat #4370, Cell Signaling, Danvers, MA); and rabbit monoclonal to synthetic Erk1/2 at 1:1000 (Cat #4695, Cell Signaling, Danvers, MA); a mouse monoclonal antibody to glyceraldehyde-3-phosphate dehydrogenase (GAPDH) from purified rabbit muscle at 1:1000 (Cat# AM4300,Life Technologies, Grand Island, NY). After overnight probing with primary antibody at 4°C, membranes were washed in PBT and incubated in the appropriate peroxidase labeled secondary against mouse (Cat #074–1806, KPL, Gaithersburg, MD) or rabbit (Cat #074–1506, KPL, Gaithersburg, MD), both at 1:10,000 for 1 hour at room temperature, then exposed to ECL Western Blot Detection Reagent (Amersham/GE HealthCare, Piscattaway, NJ) for 1 minute at room temperature. In the case where multiple antibodies were blotted on the same membrane, Restore^™^ PLUS Western Blot stripping buffer was used (Thermo Scientific, Rockford, IL) to strip the membranes. Membranes were exposed to Amersham Hyperfilm ECL (Cat # 28-9068-36, Amersham, GE Healthcare Bio-Sciences Pittsburgh, PA) and bands were then quantified using ImageJ software.

**Table 2:** Primary antibodies used for histological studies and Immunoblot.

Antibody	Dilution	Species	Company	Tissue
Erk 1/2	1:1000	Rb	Cell Signaling	Sclera (W)
pErk 1/2	1:4000	Rb	Cell Signaling	Sclera (W)
Smad 2/3	1:1000	Rb	Cell Signaling	Sclera (W)
pSmad3	1:700	Rb	Abcam	Sclera (W)
pSmad2	1:500	Rb	Cell Signaling	Sclera (W)
GAPDH	1: 1000	Ms	Ambion/Life Technologies	Sclera (W)
Thrombospondin 1	1:200	Ms	Abcam	Sclera & Retina (S)
APP	1:200	Rb	Novex/Life Technologies	ONH,Sclera,Retina (S)
Ki67	1:100	Rb	Abcam	Sclera (WM)
B-Tubulin	1:500	Ms	Covance	Retina (WM)

(W) Western blot, (S) Immunohistochemistry sections, (WM) Immunohistochemistry wholemount.

### Axonal transport blockade

Two groups of mice were exposed to elevated IOP for 3 days in one eye with bead injection, one group pre-treated with oral losartan (n = 10) and the other given water alone (n = 5). Animals were perfused with buffered 4% PFA, cryoinfiltrated with sucrose and OCT, and the posterior pole cryosectioned at 8 μm. Sections including the zone immediately through the optic nerve head were incubated in rabbit anti-amyloid precursor protein (APP) at 0.75ug/ul (Cat# 512700, Novex by Life Technologies, Frederick MD) in PBS with 0.5% Triton-X and 10% normal goat serum overnight at 4°C, washed with PBS/0.5% Triton-X, incubated with goat anti-rabbit Alexa 488 secondary antibody at 1:200 (Cat # A11008, Life Technologies, Frederick MD) and DAPI at 1:1000 (Life Technologies, Frederick MD), washed in PBS, and coverslipped with Dako mounting media (Dako, Carpinteria CA). They were evaluated in a Zeiss LSM710 confocal microscope (Carl Zeiss Microscopy, LLC, Thornwood, NY) as to the presence and degree of APP buildup at the optic nerve head area by masked observers on a scale graded either normal or 1–5+ increased labeling.

### Immunohistochemistry and confocal imaging of thrombospondin

To examine any visible upregulation of thrombospondin expression on the sclera, cryosections were prepared as in the axonal transport blockade study. Sections were incubated overnight at 4°C with mouse monoclonal thrombospondin-1 antibody (Cat# ab1832, Abcam, Cambridge, MA). The primary antibody was diluted, 1:200, in 0.1 M phosphate buffer solution (PB) and 10% blocking buffer. Blocking buffer consisted of 0.1% Triton, 2% bovine serum albumin (BSA) and 0.2% cold water fish skin gelatin (CWFG) in PB. After the overnight incubation in primary antibody solution, samples were washed in PB and then incubated for 1 hour at room temperature with goat anti-mouse AlexaFluor568 secondary (Cat # A11031, Invitrogen, Grand Island, NY) at 1:200 in PB and 10% blocking buffer. After final antibody incubation, nuclei were stained with DAPI (Invitrogen). Samples were cover slipped with DAKO mounting media (DAKO, Carpenteria, CA). Scleras and retinas were imaged with a Zeiss LSM 710 Confocal Microscope (Zeiss MicroImaging, Thornwood, NY) at the peripapillary region using a 20x objective. We used an antibody generated in mice against human thrombospondin-1 in our mouse scleral sections. To assure a lack of non-specific labeling, we took two steps: the primary antibody was incubated in goat serum containing IgG, and sections with no primary antibody were exposed in sclera tissue to secondary antibody. Since we found no detectable labeling with these sections, nor any label in control sclera, we conclude that the labeling specifically identified thrombospondin-1.

### RGC survival assay

Using a published method [36], retinas were isolated from 30 postnatal 0–3 day C57Bl/6 mice and dissociated with papain. Microglia were immunodepleted with anti-CD11b (Cat# ab1211, Abcam, Cambridge, MA) conjugated Dynabeads. The suspension of retinal cells was immunopanned on plates pre-conjugated with anti-Thy1.2 antibody (Cat# MCA02R, Serotec, Raleigh, NC) and anti-mouse IgM at room temperature (RT). After washing, RGCs were released from the plate by incubation with trypsin, counted, and seeded at a density of 3,000 per well in 384-well plates in media composed of Neurobasal (Life Sciences, Grand Island NY), NS21, Sato supplement, L-glutamine, and penicillin/streptomycin. Three sartans (kindly provided by Jung Soo Suk, Johns Hopkins University) and the kinase inhibitor, tozasertib, to be tested were dissolved in DMSO and added at the indicated concentration at the time of seeding. After a 72 hour culture at 37 degrees C, RGC viability was determined by CellTiter-Glo luminescence (Promega, Madison, WI).

### Statistical Analysis

Outcomes were compared by t test for normally distributed parameters and Mann Whitney tests for parameters failing a normality test. Data from eyes in drug treated mice were compared to fellow eye controls. Linea regression models were used to estimate the effect of treatment as well as variables such as IOP exposure (positive integral IOP, peak IOP, or average interocular difference in IOP) on RGC axon loss when comparing results of experimental glaucoma trials. In regression models, variables with the univariate p-value ≤ 0.20 were used to derive a multivariable linear model using the backward method of selection. Factors were required to have a p-value ≤ 0.10 to remain in the model. The Bonferroni or Tukey method was used to adjust for p-values for multiple comparisons among drug treatment groups.

## Results

### Normal IOP and induced IOP elevations are unaffected by losartan

**Fig 1:**
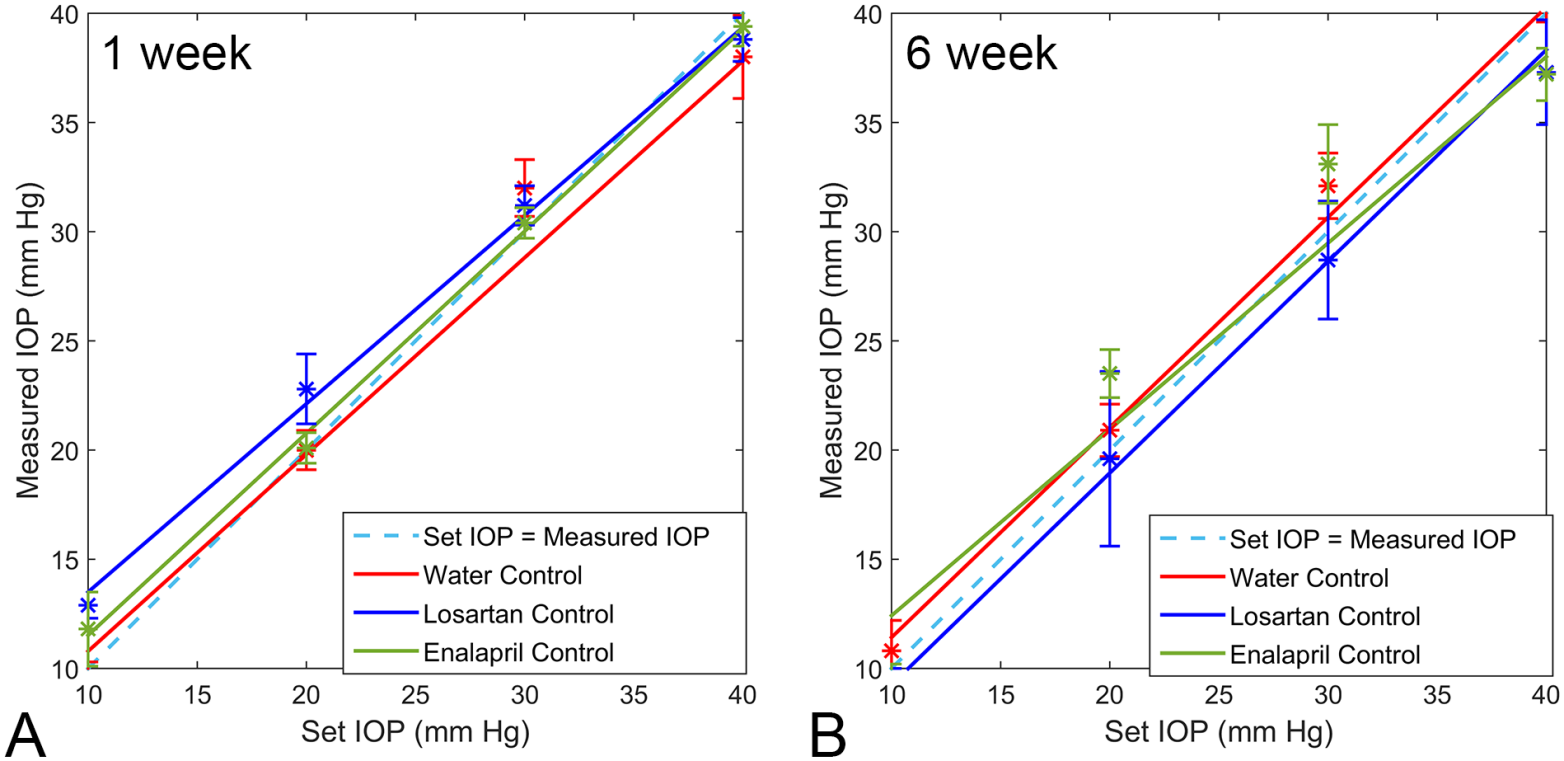
Tonolab Calibration. Calibration of Tonolab tonometer in cannulated eyes from drug-treated and water control eyes after treatment for 1 and 6 weeks. The regression lines for each group closely track with ideal calibration of manometrically-set IOP (error bars are standard error).

The calibration of the Tonolab tonometer followed the manometrically set IOP with great precision, both in control eyes and eyes that had undergone 1 or 6 week exposures to elevated IOP from bead injection, regardless of concurrent drug treatment with losartan, spironolactone or enalapril. The median Tonolab IOP at each of 5 levels of set IOP did not significantly differ among the eyes of animals that had been treated with losartan, enalapril, or no drug (water) for both glaucoma treated or untreated groups (all differences > 0.5, t test, n = 5 animals per group) (Fig 1).

There was no significant effect of losartan, spironolactone, or enalapril on IOP in either right or left eye at baseline after 2 weeks of oral dosing and prior to the production of unilateral glaucoma. Nor was there any IOP lowering indicated in the average IOP of fellow eyes that were exposed to oral drug, but not bead injection, in measurements during the 6 weeks after induction of glaucoma (Table 3).

**Table 3:** Average IOP after bead injection glaucoma.

Treatment Group	Control Eye	Glaucoma Eye	Cumulative IOP Exposure
	Mean (SD) IOP	Mean (SD) IOP	Positive Integral IOP Mean (SD)
Losartan + Glaucoma	12.2 (1.6)	16.8 (3.1)	101 (83)
Spironolactone + Glaucoma	12.6 (1.7)	16.5 (3.9)	109 (142)
Water Control + Glaucoma	12.8 (1.7)	19.9 (3.7)	162 (129)
Enalapril + Glaucoma	12.9 (1.7)	17.7 (5.4)	109 (136)
Losartan, no Glaucoma	11.7 (1.5)	11.8 (1.3)*	—

All differences among non-glaucoma eye groups and among glaucoma eye groups are statistically insignificant, with lowest p value in t tests = 0.14. Data are mm Hg with mean (standard deviation), including 7 measurements per eye, 40 eyes per group.

*Both eyes were controls in this treatment group.

The average IOP in each of 4 groups of glaucoma eyes was higher than either their fellow non-glaucoma eyes or the losartan, no glaucoma eyes (p <0.0001, t test). The overall exposure to elevated IOP as indicated by the positive integral IOP statistic was not significantly different among 4 glaucoma groups compared to each other, with statistical analysis using the Bonferroni correction for multiple comparisons (Table 3; probability value for significance with Bonferroni correction = 0.017; spironolactone vs water, p = 0.09; losartan vs water, p = 0.02; spironolactone vs losartan, p = 0.76); enalapril vs water, p = 0.07; enalapril vs losartan, p = 0.78; enalapril vs spironolactone, p = 0.98). The IOP in the glaucoma eyes with losartan was not significantly lower than sprioniolactone or enalapril groups, demonstrating that losartan did not have a protective effect by preventing IOP elevation. In regression models that compared degree of RGC axon loss among drug treated groups, the IOP data were entered as independent variables to adjust for these (non-significant) differences of IOP exposure.

### Blood pressure was significantly lowered by losartan and enalapril

Mean blood pressure was significantly lower in losartan-treated (23% decrease) and enalapril-treated mice (24% decrease) than in those drinking water alone or spironolactone-treated (p = 0.001, 0.0001, t tests, n = 20 mice; Table 4). These data confirmed our observation that the spironolactone had not remained in solution in the mouse drinking water and for that reason did not lower blood pressure. This group was thus essentially a second control group.

**Table 4:** Blood pressure data.

Treatment Group	Systolic BP	Diastolic BP
	Mean (SD)	Mean (SD)
Spironolactone	122 (17)	71 (22)
Water	119 (12)	63 (17)
Losartan	92 (17)	49 (17)
Enalapril	90 (13)	44 (9)

Data are mean (SD = standard deviation) blood pressure (BP) in mm Hg.

### Losartan prevents RGC loss in experimental glaucoma

Losartan, spironolactone, enalapril, and placebo control groups (40 animals each) underwent bead-induced glaucoma with RGC axon and body counts at 6 weeks in optic nerve cross-sections and retinal whole mounts. The median axon loss in the losartan—glaucoma group was 2.5%, which was not significantly different from no axon loss (mean 6.2 ± 23.6%, p = 0.13, t test, Table 5). By contrast, in the water—glaucoma and spironolactone—glaucoma groups, median axon loss was 26.4% and 28.3%, respectively (both p < 0.0001, t test for difference from zero loss). The median axon loss in the enalapril—glaucoma group was even greater than water-glaucoma eyes at 43% (difference from water glaucoma group, p = 0.05, multivariable model adjusted for multiple comparisons). The axon loss in the losartan—glaucoma group was significantly less than that in either the water—glaucoma, spironolactone—glaucoma, or enalapril—glaucoma groups (p = 0.03, 0.001 and p <0.0001, respectively, multivariable regression). In the regression model comparing losartan-treated to other glaucoma groups, the protective effect of losartan was significant with IOP exposure level included in the model (for losartan glaucoma vs. water or spironolactone glaucoma, multivariable model, adjusting for IOP exposure, Tukey adjustment for multiple comparisons, Table 6). The model also showed that loss of RGC was significantly related to both level of IOP elevation and to increase in axial length and width after adjustment for other variables in the model.

**Table 5:** Axon loss by treatment group.

Treatment Group	N		Control Eye	Glaucoma Eye	% Difference
Losartan + Glaucoma	38	Mean (SD)	62,331 (7,107)	56,267 (13,321)	6%
		Median	61,441	59,679	3%
Spironolactone + Glaucoma	39	Mean (SD)	63,521 (6,724)	45,148 (16,048)	29%*
		Median	64,256	45,515	28%*
Water Control + Glaucoma	40	Mean (SD)	54,823 (14,583)	41,433 (19,160)	25%*
		Median	58,276	40,868	26%*
Enalapril + Glaucoma	40	Mean (SD)	58,716 (7,097)	35,878 (20,422)	39%*
		Median	58,989	33,452	43%*
Losartan, no Glaucoma	40	Mean (SD)	62,816 (5,813)	63,371 (7,498)	—
		Median	63,957	63,247	—

* p = 0.0001, t test for difference from zero percent loss; SD = standard deviation; n = number of animals providing data per group.

**Table 6:** Multivariable model for percent axon loss.

Variable	% Axon Loss	Regression Coefficient (95% CI)	P-value	Treatments Different at Adjusted P ≤0.05^†^ ^¶^ *
Treatment			< 0.0001	G-SG (0.03)
Losartan + Glaucoma (LG)	9.9	-11.1 (-24.0, 1.8)		LG-EG (<0.0001)
Losartan only (L)	5.4	-15.7 (-31.3, -0.1)		L-SG (0.01)
Spironolactone + Glaucoma (SG)	28.2	7.1 (-5.2, 19.4)		L-EG (<0.0001)
Enalapril + Glaucoma (EG)	38.8	17.7 (5.1, 30.3)		EG-W (0.05)
Water (W) (reference)	21.1	0		L, LG, W, SG, EG
				L, LG, W^¶^
				W, SG^¶^
				SG, EG^¶^
Peak IOP Difference, mmHg (per unit increase)		0.81 (0.27, 1.35)	0.003	
% Increase in Axial Length (per unit increase)		0.97 (0.09, 1.84)	0.03	
% Increase in Axial Width (per unit increase)		-1.27 (-2.28, -0.26)	0.01	

^†^ Treatments that share a

^¶^ are not significantly different at p ≤ 0.05;

* Tukey adjustment for multiple comparisons.

Treatment with losartan, spironolactone, or enalapril without induction of glaucoma had no significant detrimental effect on RGC survival. This was shown by equal or higher mean RGC axon counts in untreated, fellow eyes of losartan, spironolactone, and enalapril mice to those in water treated mice.

### RGC cell body data

The cell body counts from retinal whole mounts labeled for β-tubulin demonstrated the same relative loss in the groups as did the axon counts. In retinas from losartan-treated glaucoma mice the RGC cell loss was 9.8 ± 9.7% compared to 33.2 ± 13.0% for the water-control glaucoma mice (p = 0.001, t test).

### Losartan decreases RGC axonal transport blockade at the ONH

**Fig 2:**
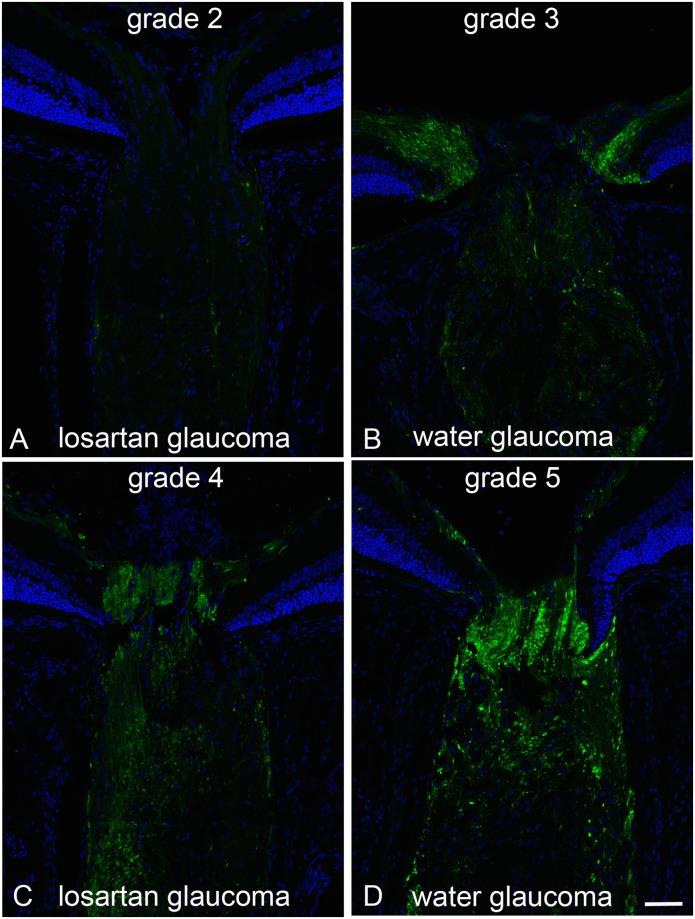
Axonal transport effects, APP labeling with losartan-treated vs water control. Immunolabeling for APP in the optic nerve head of mice treated with oral losartan (A and C) and water alone (B and D) shows axonal transport obstruction 3 days after IOP elevation. The examples show 4 of the 5 grade levels for APP accumulation (level 1, not illustrated, had no label, indicating that there was no accumulation and no background or non-specific labeling). The grades of losartan-treated eyes ranged from 2 to 4 (A and C; mean = 2.6 (sd 0.8), while water-treated eyes ranged from 3 to 5 (B and D; mean = 4.0 (sd 0.9); difference from losartan, p = 0.007, multivariable regression adjusted for IOP exposure). Bar equals 50 um.

If the beneficial effect of losartan in the glaucoma model acted by altering the effect of elevated IOP through the strain generated by IOP acting through the PPS at the ONH, it would be expected that the degree of axonal transport blockade at the ONH would be less in losartan-treated eyes. The accumulation of APP in the ONH and immediately posterior optic nerve was clearly evident after 3 days of IOP elevation in controls. There was significantly less axonal transport block in losartan-treated glaucoma eyes compared to water-treated glaucoma eyes. The masked grading on a 1–5 (mild to severe) quantification scale was 4.0 ± 0.9 in water glaucoma and 2.6 ± 0.8 in losartan glaucoma eyes (p = 0.007, multivariable regression, adjusted for IOP exposure; Fig 2).

### Losartan does not protect RGC after optic nerve crush

**Fig 3:**
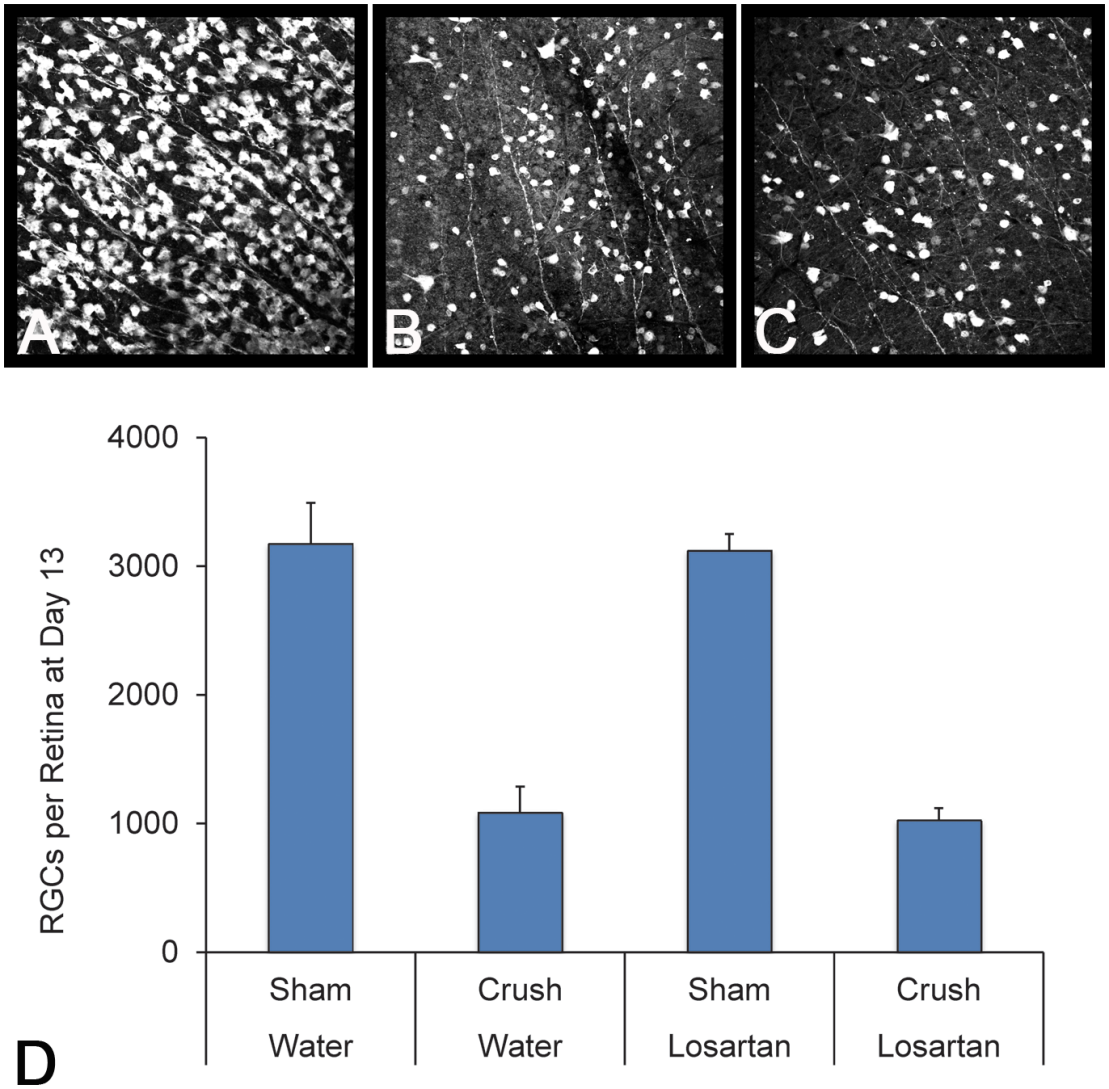
Losartan is not neuroprotective in a crush model of axon injury. Images of RGCs stained with Sncg from the sham losartan group (A), crush water group (B) and crush losartan group (C), showing no difference in RGC survival from losartan treatment from axon crush. The graph shows all four groups, with the sample sizes for Sham Water, Crush Water, Sham Losartan and Crush Losartan groups n = 9, 9, 10 and 13 respectively.

Since losartan had demonstrated neuroprotection in the glaucoma model, we wished to test whether its protective effect was operative for any axonal injury to RGC, or was specific to the mechanism of injury related to elevated IOP. To answer this question, we turned to a different rodent model of optic neuropathy in which the optic nerve is mechanically crushed, representing an axonal injury, but not from high IOP. Again, mice were pretreated with losartan or vehicle for two weeks, and then subjected to forceps crush. After an additional 13 days, both losartan and control groups showed a similar reduction in RGC cell numbers in the retina. There was a significant loss of RGC in both the losartan-treated and water-treated mice at 13 days after crush injury. There was no significant difference between the two groups of crush animals (p = 0.91, n = 9 and 10 mice, respectively; Fig 3).

### Losartan does not prolong RGC life in dissociated culture

We further studied the neuroprotective effect of sartans on RGC by evaluating their potential benefit in prolonging RGC life in dissociated culture. If sartans exert a protective effect on RGC bodies, this might be evident as a prolongation of RGC survival in an established culture system [36]. For this comparison, losartan was not used, as its maximum action occurs only after processing in the liver to a first stage metabolite. The survival of dissociated mouse RGC in culture at 72 hours was compared among irbesartan, valsartan, telmisartan, and tozasertib. As expected, tozarsertib, an inhibitor of dual leucine zipper kinase, robustly increased the number of surviving RGCs. In contrast, none of the sartans differed significantly from controls without added drug, despite being tested over a broad dosage range.

### Retinal thickness is not affected by losartan treatment

In epoxy-embedded sections of retina, the average thicknesses of the inner and outer nuclear layers (INL, ONL) were measured in masked fashion. The fellow eyes (non-glaucoma) of losartan-treated and water-treated mice did not differ significantly in the average thickness of either retinal layer (INL: losartan: 31.2 ± 3.7 μm, water: 34.9 ± 2.4 μm, p = 0.1; ONL: losartan: 43.7 ± 2.0 μm, water: 44.7 ± 1.7 μm, p = 0.4, t tests). Likewise, in glaucoma eyes, the average INL and ONL thicknesses did not significantly differ between losartan-treated and water controls (INL: losartan: 33.3 ± 3.0 μm, water: 35.4 ± 3.0 μm, p = 0.3; ONL: losartan: 44.9 ± 3.2 μm, water: 47.5 ± 3.0 μm, p = 0.2). Similarly, the glaucoma compared to fellow eye differences within treatment groups did not significantly differ in INL or ONL thickness (all p values > 0.2).

### Scleral alterations typically found in experimental glaucoma are modified by losartan

#### Axial length and width

After 6 weeks of induced IOP elevation, there was a significant axial length increase of 5–7% in treated eyes of all 4 glaucoma groups compared to the untreated eyes and to the losartan—no glaucoma eyes, with mean control axial length varying from 3.64 to 3.70 mm and glaucoma eyes varying from 3.86 to 3.94 mm (all group differences between glaucoma and control eyes and between glaucoma and losartan—no glaucoma eyes significant, p < 0.002 or less, t tests; Table 7). Similarly, the ocular width significantly increased in treated compared to fellow untreated eyes by 4–6%. The axial length and width increase in the losartan—glaucoma group did not differ significantly from the other glaucoma groups (all p = 0.5, t tests). There was no change in ocular length or width due to losartan treatment alone without glaucoma. In enalapril animals, the non-glaucoma fellow eyes were significantly smaller than the non-glaucoma eyes in the other 4 groups, and likewise the glaucoma eyes were smaller, even though their proportionate increase after chronic IOP exposure (4% and 7%) was equal to the other groups.

**Table 7:** Axial length and width.

Treatment Group	Control Length (mm)	Glaucoma Length (mm)	Control Width (mm)	Glaucoma Width (mm)
Losartan + Glaucoma (n = 38)	3.65 (0.50)	3.94 (0.21)	3.64 (0.09)	3.82 (0.23)
Spironolactone + Glaucoma (n = 39)	3.70 (0.09)	3.87 (0.25)	3.62 (0.09)	3.78 (0.21)
Water Control + Glaucoma (n = 40)	3.66 (0.10)	3.93 (0.24)	3.59 (0.09)	3.79 (0.21)
Enalapril + Glaucoma (n = 40)	3.64 (0.13)	3.86 (0.23)	3.47 (0.15)	3.63 (0.22)
Losartan, no Glaucoma (n = 40)	3.67 (0.10)	—	3.62 (0.10)	—

Data are mean (standard deviation) in mm.

#### Scleral thickness

In 20 mice treated with losartan, but without induction of elevated IOP, the PPS in unfixed tissue was thinner than in animals not exposed to losartan (Table 8). The fellow eyes in the losartan—glaucoma group were 7.6% thinner than the fellow untreated eyes in the water—glaucoma group and 17.1% thinner than the fellow eyes of spironolactone—glaucoma (losartan—glaucoma fellow vs water—glaucoma fellow or vs spironolactone—glaucoma fellow, p = 0.0017 and p < 0.0001, respectively; t tests). Similarly, the mice treated with losartan but without glaucoma in either eye had 9.5% thinner PPS than the combined fellow controls of the water—glaucoma and spironolactone—glaucoma groups (p = 0.0001, t test).

**Table 8:** Unfixed Peripapillary Thickness.

Parameter	Losartan + Glaucoma		Losartan only	Spironolactone + Glaucoma		Water + Glaucoma	
	Glaucoma	Control	Control	Glaucoma	Control	Glaucoma	Control
Mean (SD)	44.3 (3.4)	42.4 (3.8)	43.9 (4.0)	47.2 (5.8)	51.1 (7.2)	42.9 (4.0)	45.8 (2.4)
	Gl-C^†^	—	LE—RE*	Gl-C^†^	—	Gl-C^†^	—
Mean Difference (SD)	1.9 (4.5)	—	-0.5 (3.7)	-4.0 (6.7)	—	-2.9 (5.0)	—

Data are mean (standard deviation), in microns. The treatment group sizes were: Losartan + Glaucoma (n = 20), Losartan Only (n = 20), Spironolactone + Glaucoma (n = 20) and Water + Glaucoma (n = 19).

^†^Glaucoma minus Control.

* Both are control eyes in this treatment group.

In eyes exposed to elevated IOP in the water—glaucoma and spironolactone—glaucoma groups, the PPS in unfixed tissue was thinner than in the fellow control eyes (3% thinner, water—glaucoma vs. control (p = 0.02); 4% thinner, spironolactone—glaucoma vs. control (p = 0.03, t test of paired glaucoma to fellow eye differences). In the losartan—glaucoma group, however, the PPS was 2% thicker in the higher IOP eye than in fellow eye controls (p = 0.07, paired t test). The glaucoma compared to fellow eye thickness difference in the water and spironolactone glaucoma groups was significantly different from that of the losartan—glaucoma group (p = 0.003 and 0.002, respectively).

Compared to unfixed sclera, the scleral thickness after fixation and embedding in epoxy showed that in the 15 mice (30 eyes) treated with losartan alone, fixed PPS thickness was significantly (11%) thicker than the fellow, untreated eyes of controls (p = 0.005, n = 30 eyes per group, t test; Table 9). In eyes exposed to elevated IOP in the water and in the spironolactone groups, the fixed PPS was thicker than in their fellow control eyes (p = 0.04 and 0.04, paired t tests, n = 15 per group, Table 9). By contrast, the losartan treated eyes with glaucoma exposure had no increase in the fixed PPS compared to their fellow eyes (p = 0.57, paired t test).

**Table 9:** Fixed Peripapillary Thickness.

	Losartan + Glaucoma		Losartan only	Spironolactone + Glaucoma		Water + Glaucoma	
	Glaucoma	Control	Control	Glaucoma	Control	Glaucoma	Control
Mean (SD)	36.8 (6.2)	35.2 (6.9)	37.8 (4.7)	38.9 (5.8)	34.7 (5.5)	36.9 (4.6)	33.2 (5.9)
	GL-C^†^	—	LE—RE*	GL-C^†^	—	GL-C^†^	—
Mean Difference (SD)	1.6 (10.2)	—	-0.8 (5.6)	6.5 (9.8)	—	3.8 (6.1)	—

Data are mean (standard deviation), in microns.

^†^Glaucoma minus Control.

* Both are control eyes in this treatment group.

The number of scleral fibroblasts was quantified in whole mounted sclera from randomly selected fields, comparing control, fellow eyes to glaucoma eyes with and without losartan treatment 6 weeks after IOP elevation (from peripapillary to mid-equatorial sclera, 5 eyes per group). The fibroblast density increased significantly in non-losartan-treated glaucoma sclera (450 ± 54 fibroblasts/0.14 mm^2^) compared to their controls (237 ± 37, p < 0.0001, t test) after 6 weeks of glaucoma. By contrast, the density increase in losartan-treated glaucoma sclera was much smaller and not significantly different from fellow—controls (381 ± 101 vs. 308 ± 27, p = 0.15, Mann Whitney test). These measurements were made in whole-mounted sclera and thus not subject to the more substantial tissue shrinkage of embedded tissues. We examined for this study only sclera at 6 weeks after IOP elevation, and we previously showed that most cell division induced by IOP elevation occurs during the first week. Thus, it was expected that we would find relatively few Ki67 positive fibroblasts; indeed, only 1–2% of sclera fibroblasts were positive. Therefore, we did not have sufficient numbers of dividing cells to test for differences between groups. Interestingly, of the Ki67 positive cells, 71% were found in the peripapillary zone (R1), far greater than would be expected by chance alone for the 5 areas studied.

#### Inflation testing

**Fig 4:**
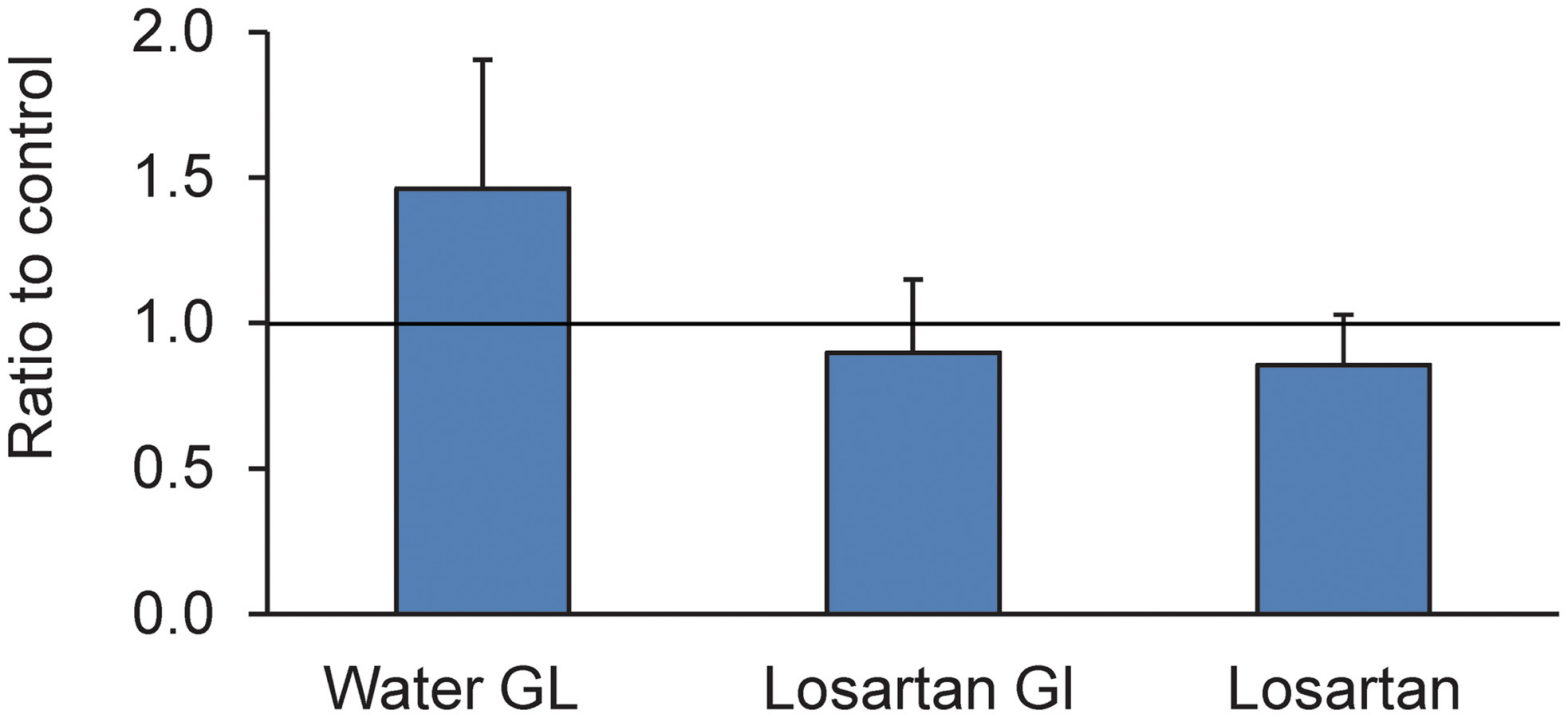
The effect of losartan on creep rate in ramp—hold inflation studies. The creep rate during a 30 minute period at 30 mm Hg for losartan- and water-treated glaucoma eyes was compared to control eyes (represented by the horizontal line drawn at the ratio of one), along with losartan without glaucoma compared to control eyes as a ratio. Losartan treatment alone and losartan-glaucoma eyes had no difference in creep rate from controls, but water-treated glaucoma eyes had a greater creep rate than controls.

Losartan-treated mice had different inflation behavior than water-treated controls after exposure to elevated IOP (Fig 4). In each case, the data as presented are the ratios of experimental to control creep and are thus unitless values. In losartan-glaucoma eyes the circumferential strain creep ratio was 0.90 ± 0.25, while in water-treated glaucoma eyes it was 1.46 ± 0.44 (p = 0.002, n = 12, 10 per group, t test). Thus, the ratio in water-glaucoma eyes significantly exceeded one (p < 0.0001), while the losartan-glaucoma ratio did not differ from one (p = 0.3), nor did the losartan without glaucoma eyes compared to water-treated controls differ significantly from a ratio of one (0.85 ± 0.22, p = 0.3).

**Fig 5:**
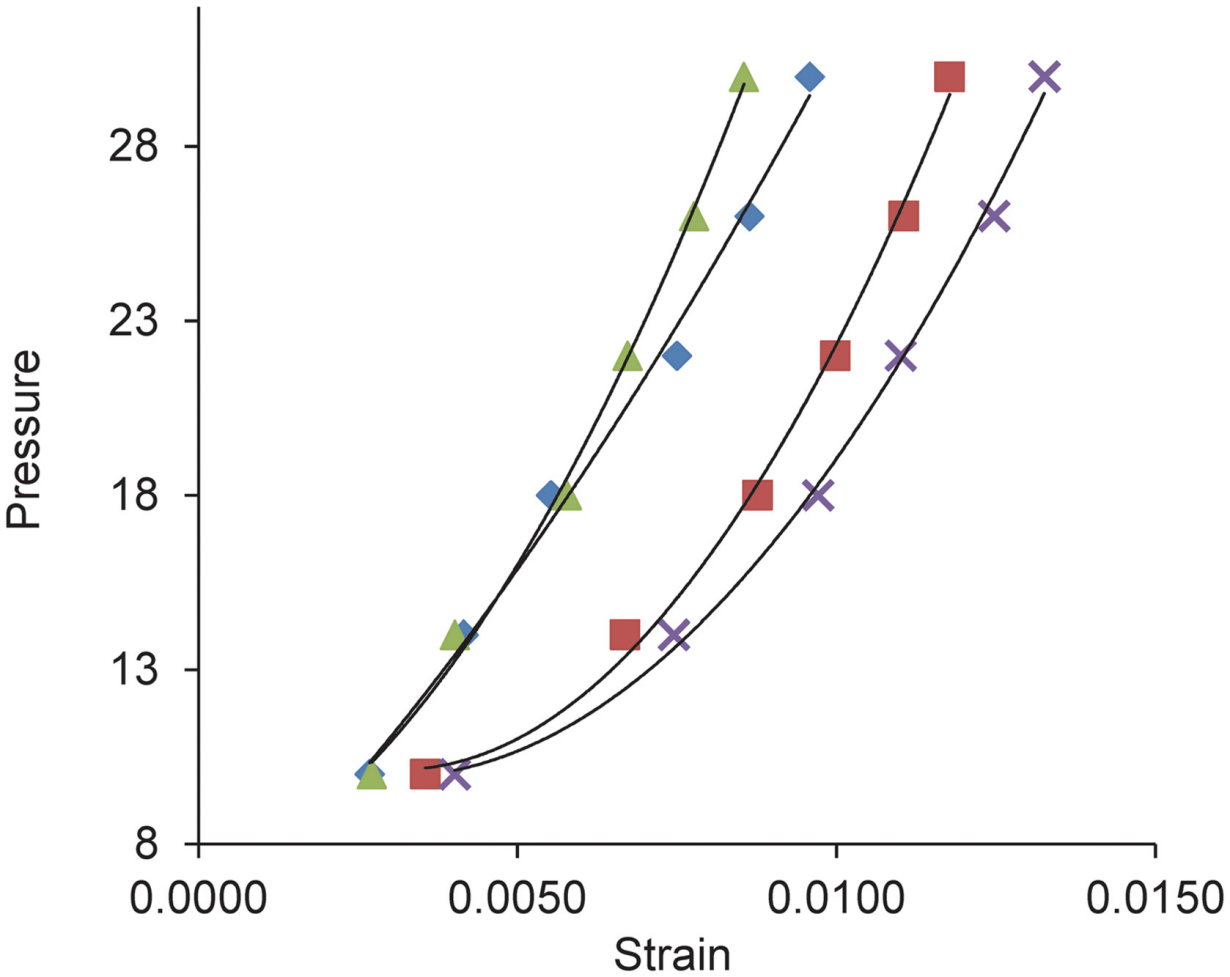
Pressure—strain estimates in load—unload protocol for losartan and control groups. Pressure—strain relationships in load—unload inflation tests show steeper (stiffer) curves for glaucoma eyes of losartan (triangle) and water-treated (diamond) compared to their respective fellow, control eyes (x and square, respectively). Y axis = inflation pressure in mm Hg.

In rapid inflation to 30 mm Hg (load—unload) data, losartan treated eyes that were exposed to elevated IOP for 6 weeks were significantly stiffer than their fellow eye controls in all regions in the circumferential direction and in the temporal meridional direction (p values in 4 scleral regions from ONH to mid-sclera ranged from 0.03 to 0.008; Fig 5). There was a 40% decrease in strain in losartan-glaucoma eyes compared to controls. In the water—glaucoma group, the glaucoma eyes were also stiffer, but less than in the losartan-glaucoma group, and the increase in stiffness did not reach statistical significance (all p values > 0.05). Losartan treated mice that were not exposed to glaucoma had no significant difference in any pressure—strain estimates from the fellow control eyes of water—glaucoma group (all p values > 0.05, t tests). Among those eyes selected for inflation testing, there was no significant difference in the IOP exposure of losartan and water glaucoma eyes (p = 0.8).

### Losartan modifies elements of TGFβ-related signaling in sclera

**Fig 6:**
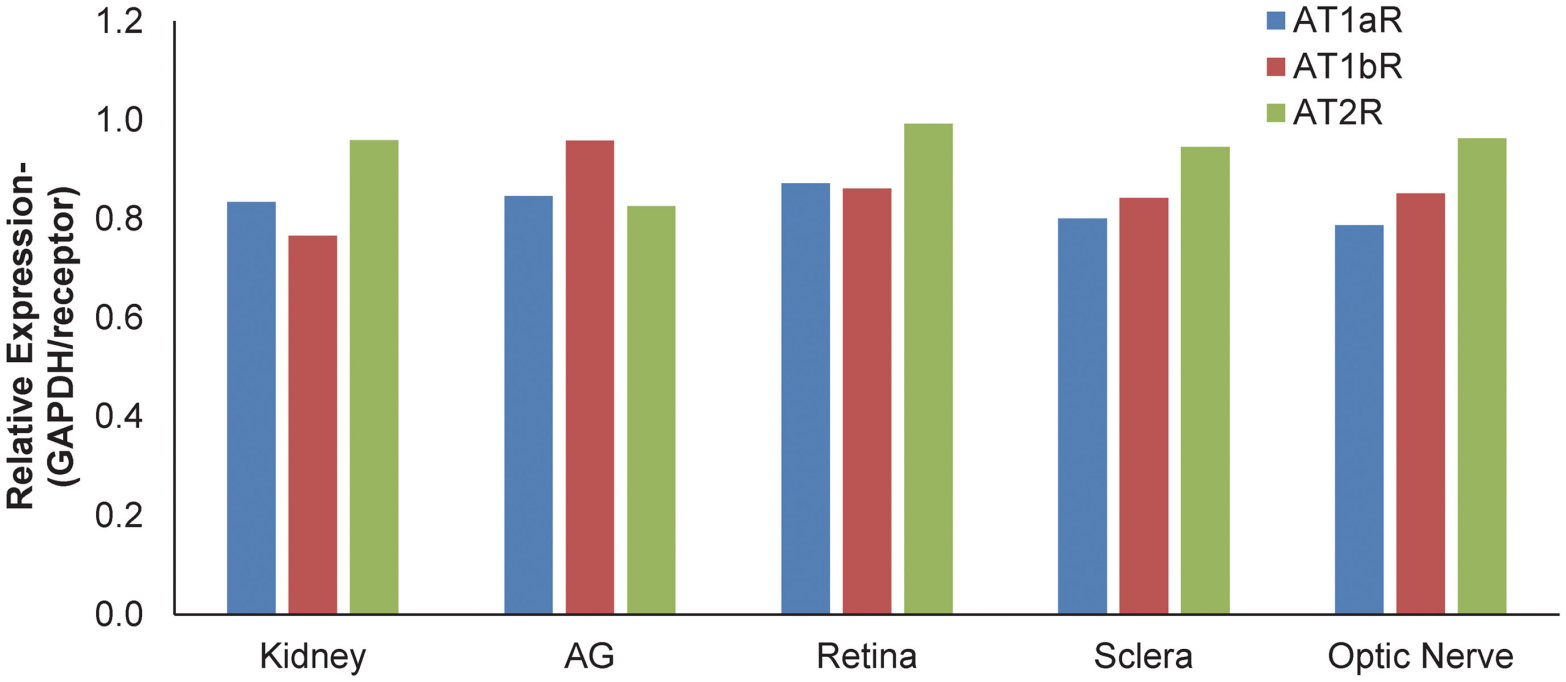
RTPCR of angiotensin receptor expression. Graph plots the relative expression of five tissues tested for the presence of AT1a, AT1b, and AT2 receptors- kidney, adrenal gland, retina, sclera and optic nerve from a CD1 mouse. To help normalize the results, a value was calculated by dividing the GAPDH primer treated tissue value by the mean quantification cycle (Cq) value for AT1A, AT1B, or AT2 primer tissue (relative expression). A lower value than 1 indicates a lower value for that receptor in tissue compared to GAPDH.

We hypothesized that the neuroprotective effect of losartan acted by inhibiting the AT1R in sclera. However, the presence of the angiotensin receptors had not been previously shown for sclera, as it had been for many tissues, including the retina and choroid. Quantitative RTPCR showed that the relative expression of AT1aR, AT1bR, and AT2R (normalized for expression of GAPDH) was similar in adrenal gland, kidney, retina, optic nerve and sclera in CD1 mice (Fig 6).

**Fig 7:**
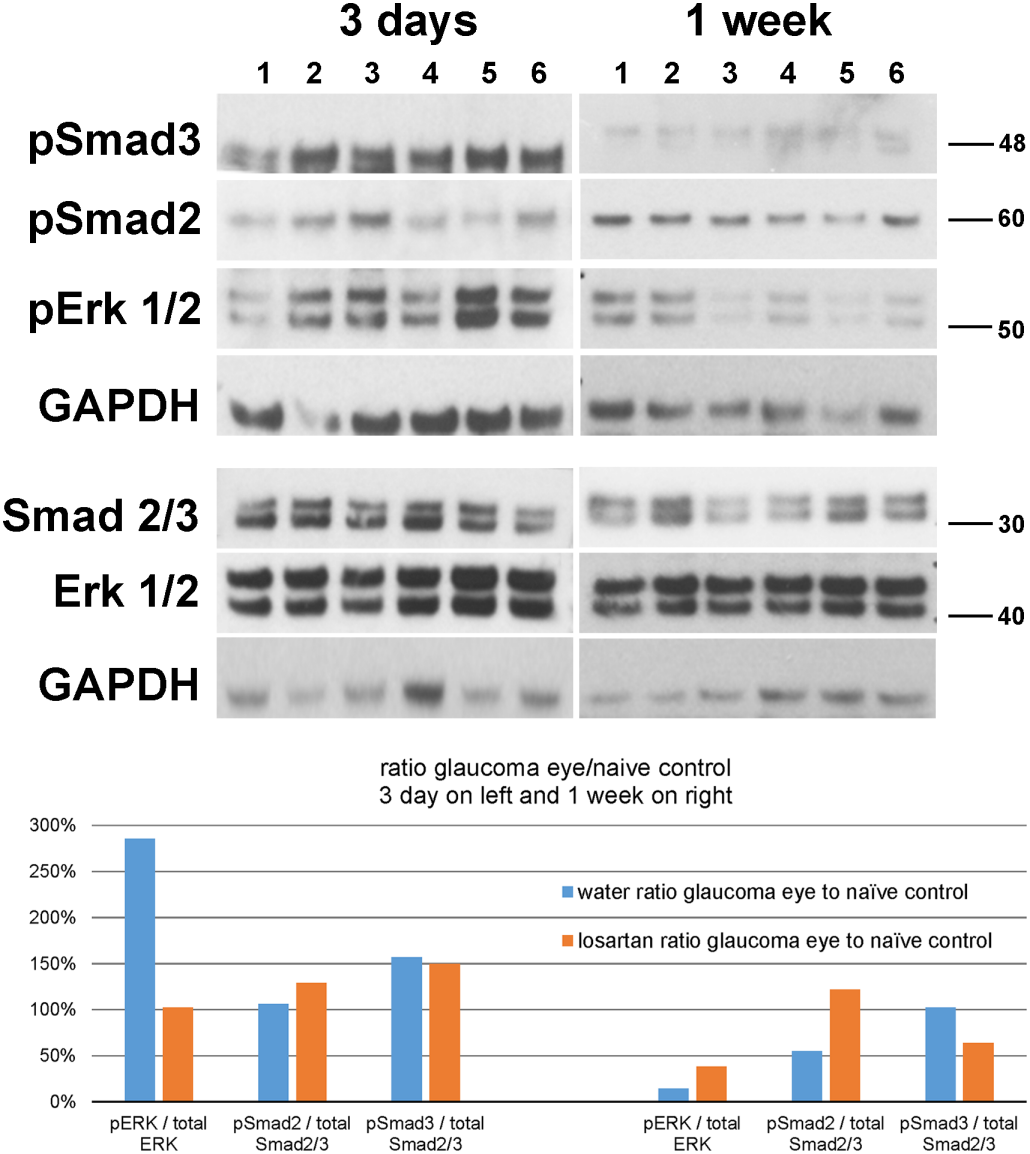
Western blots comparing losartan and water groups. Immunoblots for pERK1/2, ERK1/2, pSmad2, pSmad3, Smad2/3 and GAPDH for 12 scleral specimen groups. (1) water control; (2) losartan control; (3) fellow eyes of water-treated glaucoma; (4) fellow eyes of losartan-treated glaucoma; (5) water-treated glaucoma; and (6) losartan-treated glaucoma. Graphs show quantification by densitometry of blots, indicating the ratio of the activated molecules to the total amount of that molecular species: pERK/total ERK, pSmad2/total Smad2/3, and pSmad3/total Smad2/3. The comparisons are among all types of glaucoma eyes compared to naïve control eyes at 3 days (left group) and 1 week (right group) after glaucoma. Water-glaucoma group in blue shows 3-fold elevation in pERK/ERK ratio, while no difference from control is seen in losartan-glaucoma eyes. At one week, both water and losartan glaucoma eyes have substantial reduction in pERK/ERK ratio compared to controls. Very mild increases are seen in pSmad ratios in either water or losartan groups. See S1 Fig for original western blots.

Immunoblots for the Thr202/Tyr204 dual-phosphorylated, activated form of ERK (pERK), showed a significant rise in pERK in water-treated glaucoma eyes compared either to bilaterally naïve controls or fellow eyes, supporting the hypothesis that stimulation of the AT receptors is involved in the response to glaucoma (Fig 7). At 3 days after IOP elevation, the pERK/GAPDH ratio of water-glaucoma eyes was 0.98, while in losartan-glaucoma eyes it was 0.68, as would be expected from inhibition of AT1R. The IOP increase in the water-treated and losartan-treated glaucoma eyes did not significantly differ at either 3 days or 1 week of glaucoma (p = 0.22, 0.38, t tests). These data were generated by pooling 10 scleras for each group, as individual mouse scleras do not contain sufficient protein to perform the analysis as individual eyes. To confirm the Western finding, two additional runs of pooled scleral comparison were performed. The first confirmed that in 10 water-glaucoma eyes the pERK/GAPDH ratio was elevated compared to controls with a ratio of 1.39, nearly twice the ratio for losartan-glaucoma. In a third set of eyes, with 6 scleras in each group, the ratio of pERK to total ERK, pSmad2/total Smad2/3 and pSmad3/total Smad2/3 were compared (Fig 7). As in the first two analyses, water-treated glaucoma eyes had nearly 3 times higher ratio of pERK/ERK compared to non-glaucoma controls, compared to the losartan-treated glaucoma eyes, whose ratio of nearly 1 showed a lack of response to glaucoma in this activity. Interestingly, at 1 week after glaucoma induction, both groups had far lower than control ratios of pERK/ERK (Fig 7).

At 3 days and 1 week after IOP elevation, the amounts of pSmad2 and pSmad3 and their ratios to total Smad2/3 were increased but only to a maximum of 50% greater than control for the higher of the two (Smad3; (Fig 7).

**Fig 8:**
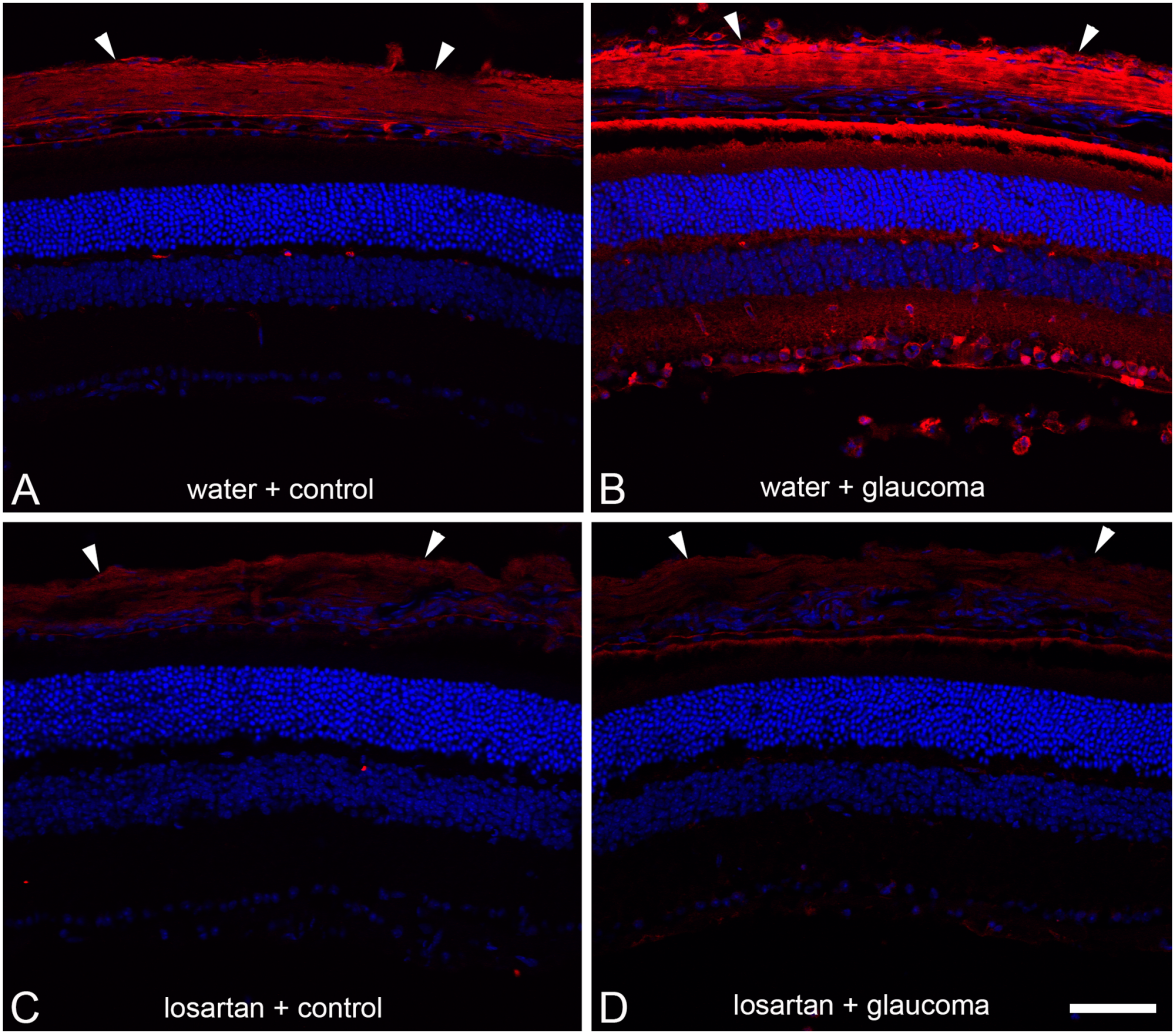
Immunohistochemical labeling for thrombospondin 1. Immunostaining for thrombospondin-1 shows mild scleral label (arrowheads) in water-treated fellow eye (A) and dramatically increased label in both sclera and retina of water-glaucoma eye (B). Thrombospondin 1 label was less in sclera and retina of both the losartan fellow eye (C) and losartan-glaucoma eye than the corresponding water controls (D, scale bar = 50μm; DAPI counterstain).

Immunolabeling for thrombospondin 1 was compared in masked fashion in 24 cryosections of 6 glaucoma eyes compared to 6 fellow eye controls. In every case, the observer correctly identified the glaucoma eye due to greater thrombospondin label in both the retina and sclera compared to the control eye. In a masked, quantitative grading on a 5 level scale, there was less retinal and scleral label in the losartan-treated than in the water-treated eyes, but the differences did not achieve statistical significance with the modest sample tested (Fig 8).

## Discussion

The present results strongly indicate that losartan, an inhibitor of the AT1R receptor, is neuroprotective for RGC in the mouse model of glaucoma. The beneficial effect was very substantial, nearly eliminating any detectable loss of RGC caused by experimentally elevated IOP, compared to 3 different treatment groups exposed to glaucoma. This represents the third result in which an alteration in the sclera of mice has influenced the degree of RGC death in murine glaucoma. In one prior publication, we demonstrated that a mouse strain with induced mutation in collagen 8α2 and altered scleral anatomy and physiology had dramatically reduced susceptibility to glaucoma damage [16]. Second, exposure of the sclera to glyceraldehyde to increase scleral fiber cross-linking led to greater RGC death than in untreated mice with similar elevated IOP exposure [17]. Here, we present substantial evidence that losartan protected RGC by affecting the scleral response to elevated IOP. It is striking that losartan’s beneficial effect was present despite a significant lowering of the perfusion pressure to the eye. In human glaucoma, lower perfusion pressure is significant risk factor for incidence and prevalence of disease [37]. This points away from a vascular mechanism as the pathway utilized by losartan treatment to benefit RGC survival. A previous publication with oral delivery of a related AT1R inhibitor, candesartan, found reduced RGC loss in 6 treated rats [25]; however, these investigators found no blood pressure lowering from the dose of candesartan used. Furthermore, they did not study the mechanism by which candesartan exerted its possible beneficial effect. They attempted to label retinal tissues with antibodies to AT1R receptor, showing positive reaction in the RGC layer, but no double labeling was performed to indicate that the putative AT1R labeling was within RGCs. In fact, the antibodies they used are now known to fail to identify AT1R specifically [34]. In other past reports, this may affected conclusions regarding the location of AT1R location in retina of rat [38] and human eyes [39,40], immunolabel has been seen from iris/ciliary body to retinal neurons, glia and blood vessels, and labeling of RGC was inconsistent. Binding of angiotensin II to retinal tissue, including pericytes, has also been reported [41]. AT1R gene expression by RTPCR was reported in retina, iris/ciliary body, and choroid of adult rats [42]. We found AT1R and AT2R expression in sclera by RTPCR, confirming the possibility that the effect of sartans is potentially on scleral fibroblasts.

We propose that systemic delivery of losartan preserved RGC in experimental glaucoma through effects on the sclera and/or on astrocytes of the ONH. Direct evidence that the protection by losartan was through modification of the scleral response to IOP elevation was the fact that there was reduced axonal transport blockade in losartan-treated glaucoma optic nerve head compared to controls. The stress of IOP is delivered to RGC axons at this site and a reduction of axonal injury provides strong evidence that losartan acted by altering IOP-generated stress in the PPS. Both RGC cell bodies and their axons were preserved to the same degree after losartan treatment, adjusted for IOP exposure in the glaucoma model. This is consistent with a mechanism in which the initial injury to the RGC axon at the ONH is prevented by losartan. If its benefit acted at a time later than initial axon injury, for example by altering the death process at the RGC body, then it would be more likely that cell bodies would survive, but axons undergo Wallerian degeneration. Further evidence that the effect was specific to axonal injury delivered via the sclera was the failure of losartan and other sartans to protect RGC from optic nerve crush injury or to prolong the life of dissociated RGC in culture.

We present several additional lines of evidence that the scleral response to glaucoma is favorably altered by losartan. First, the relevant receptor on which losartan acts, AT1R, is expressed in the sclera. A related molecule, candesartan, blocks the stretch-induced response of cardiac myocytes by inhibiting the AT1R [43]. Second, losartan treatment altered the biomechanical response of the sclera, maintaining its creep rate in the normal range on *in vi*tro inflation testing. By contrast, the creep rate in non-losartan glaucoma eyes increased. Third, losartan-treated eyes did not show the vigorous increase in scleral fibroblast density found in control glaucoma eyes, particularly in the PPS. Fourth, losartan inhibited the increase in pERK among scleral fibroblasts that was found in non-sartan-treated glaucoma eyes. The relative increase in pSmad2 and 3 in mouse glaucoma sclera was much more modest and did not differ between losartan-treated and water-treated eyes. This is consistent with the mechanism by which losartan was beneficial in a mouse model of Marfan syndrome [44]. In that model, oral losartan decreased aneurysm formation, decreasing pERK levels and its protection required intact AT2 signaling. In the Marfan mouse, enalapril, which limits signaling through both AT1R and AT2R, was not as beneficial as losartan, and our data show enalapril actually made glaucoma worse. Both drugs attenuated canonical TGFβ signaling through pSmads, but losartan inhibited TGFβ-mediated activation of pERK, by allowing continued signaling through AT2. This suggests that the non-canonical pathway downstream from the AT receptors, including actions of TGFβ, is operative in losartan’s beneficial effect in the sclera of glaucomatous mice.

Research on corneal fibroblasts supports the importance of TGFβ signaling in control of ocular extracellular matrix remodeling. AT1R is expressed by corneal myofibroblasts and anterior ocular fibroblasts respond to angiotensin II stimulation with increased AT1R activity and fibrogenesis [45]. Losartan attenuates the effects of angiotensin II among corneal myofibroblasts in rabbit eyes, altering apoptosis, NF-κB activity, and TGFβ production [46]. The renin-angiotensin system (RAS) activates TGFβ and local RASs have been identified in several tissues, including the eye [47,48,49]. Angiotensin II (AngII) type 1 receptors (AT1R) have been previously identified in the retina, the retinal pigment epithelium (RPE) and the choroid [50,51,52,53], and the present data show they are expressed in sclera. Blood flow to the ONH through the posterior ciliary arteries is responsive to Ang II [54,55]. RAS activity is initiated when renin cleaves angiotensinogen into Ang I, which is converted by angiotensin converting enzyme (ACE) to Ang II [56]. AngII activates AT1R and angiotensin II type 2 (AT2R) receptors and ACE inhibitors block its action on both AT1R and AT2R. The activation of AT1R produces vasoconstriction, fibrosis, inflammation and proliferation, while stimulation of AT2R has the opposite effects on these processes. In the eye, AT1R activation may be involved in the pathogenesis of diabetic retinopathy [57,58], neovascularization in ischemic retinopathy [59,60,61], age-related macular degeneration [62,63,64] and scarring after glaucoma surgery [65]. Signaling through AT1R upregulates expression of thrombospondin 1, a potent activator of TGFβ [66,67,68,69] in mouse glaucoma sclera showed increased thrombospondin, a potent activator of TGFβ, which was somewhat less in losartan-treated than in water-treated glaucoma sclera.

It is possible that sartans have some additional protective effects for RGC by acting in the retina directly on RGC bodies or through other neurons or glia. In cultured retinal explants, sartans somewhat delay RGC death [70,71] a mechanism suggested to involve reduction in free radicals. On the other hand, we found no increase in RGC survival in dissociated culture using 3 sartans over a wide range of concentrations. Furthermore, if the losartan benefit was acting on the RGC at the soma, one would expect that it would have a protective effect with axon crush. There are past clear indications that crush and experimental glaucoma show similar features and some compounds exhibit protection in both models [54]; however, this was not the case for sartan neuroprotection. There is other evidence that the retina is not the site of action for RGC neuroprotection by losartan. Infusion of angiotensin II in rats led to increases in vascular endothelial growth factor (VEGF), which is protective for RGC loss in the rat glaucoma model [72,73,74] losartan treatment blocks the increase in VEGF [75], argues against a direct neuroprotective effect of AT1R inhibition by losartan on RGCs themselves. Furthermore, a similar AT1R inhibitor, valsartan, failed to have neuroprotective effects in a rat ocular ischemia model and even evidenced inner retinal toxicity on oral dosing at higher levels [76]. While there is abundant evidence for increase TGFβ activity in the ONH of human glaucoma eyes, present data suggest that, on the contrary, TGFβ activity in the glaucomatous retina actually declines [77].

We found no evidence that the beneficial effect of losartan was due to a lowering of IOP. It has been reported that topical application of AT1R inhibitors in rat [78] and monkey [79] eyes decreased IOP. One report found lower IOP after systemic losartan administration in humans [80]. However, we found no change in IOP with systemic dosing of losartan in the mouse. Nor was the IOP elevation after bead injection significantly different among the 4 groups of mice, including that receiving losartan. The calibration of the Tonolab tonometer was unaffected by either losartan treatment alone or the induction of axial enlargement and other changes in the cornea and sclera with experimental glaucoma. Despite the fact that systemic blood pressure was lower in the losartan-treated mice, the decrease in calculated perfusion pressure was associated with greater RGC survival. By contrast, the lowered perfusion pressure associated with enalapril treatment led to greater RGC death than untreated controls. To our knowledge, this is the first report of greater RGC loss with blood pressure reduction in an animal glaucoma model, though the association of greater open angle glaucoma prevalence at lower perfusion pressure is well-documented in humans [37]. We did not measure actual retinal or ONH blood flow in this experiment, which is technically challenging in mice. It is reported that angiotensin II is not a major determinant of cerebral and ocular blood flow in vivo, though angiotensin II increases systemic blood pressure and pulse pressure [81].

The role of scleral biomechanics in glaucoma pathogenesis is more complex than simple changes in baseline state of the tissue, and more likely derives from alterations in the dynamic tissue response to elevated IOP in this model. The present data point to features of the scleral thickness of losartan-treated eyes without glaucoma exposure that were similar to controls that had experienced elevated IOP. This may indicate a beneficial pre-conditioning effect of the losartan treatment. Furthermore, losartan-treated eyes that underwent glaucoma developed steeper stress—strain curves with rapid loading as did controls, but had significantly lower creep rates on sustained IOP elevations. This seems compatible with the maintenance of some aspects of the normal scleral state in losartan-glaucoma eyes. Consistent with this hypothesis, losartan treated glaucoma eyes also failed to undergo increased fibrosis and loss of matrix as did control glaucoma eyes. Each of these findings supports dynamic inhibition of fibrous tissue remodeling related to the TGFβ pathway as a beneficial mechanism of losartan in this model. Losartan treatment without induction of chronic IOP elevation did not change the baseline pressure—strain response or creep rate of the sclera, and left the creep rate within control values after sustained IOP elevation. This was significantly different from the water-treated glaucoma eyes, and from the biomechanical response of eyes treated with subconjunctival glyceraldehyde in a previous study. We propose that the rapid load—unload behavior of sclera becomes stiffer in most eyes after chronic IOP elevation due to the uncrimping of collagen fibrils and the reorientation of scleral lamellae, as detailed in a prior publication [13]. These effects could be viewed as passive responses to the stress of IOP, occurring whether the eye is exposed to sartan or not. This type of behavior would not result from dynamic cellular responses. On the other hand, the maintenance of normal creep rate in losartan-treated eyes, the differences in fresh and fixed scleral thickness and the reduction in fibroblast proliferation in losartan-treated eyes represent altered remodeling of the sclera and may be critical to the beneficial effect of the sartans on RGC survival.

The detailed effects of losartan treatment without induction of glaucoma on the mouse sclera included modest decrease in fresh scleral thickness and increases in scleral thickness in fixed—epoxy embedded sections. These changes most likely indicate that losartan led to an increase in collagenous fibrils and less glycosaminoglycan content at baseline. Control eyes that experience chronic IOP elevation have fibril increase and matrix loss similar to that caused by losartan alone without glaucoma. Thus, losartan-treated eyes that undergo chronic IOP elevation begin at baseline with features already in place that characterize the post-glaucoma state in non-losartan-treated eyes. Unlike non-losartan treated eyes, the losartan pre-treated sclera that is exposed to higher IOP undergoes no further change in fixed scleral thickness (i.e. in fibril content) and has an actual increase in fresh scleral thickness—indicating an expansion of its matrix component. Protective effects of losartan treatment have also been seen in combat injury-related muscle remodeling and disuse atrophy of muscle [27]. In addition, losartan acts to decrease fibrosis in paraquat-induced pulmonary fibrosis [82].

This work has limitations that may temper its conclusions. Without more detailed examination of the scleral content, conclusions about the effects of losartan on scleral structure require confirmation. The inflation method used has been shown to be reproducible and to correlate with alterations in scleral state. However, it is most reflective of changes in scleral behavior that are detectable on the surface of the sclera. Newer methods are in development to study not only the full thickness of PPS, but also the biomechanical behavior of the ONH astrocytes that constitute the equivalent of the lamina cribrosa of the primate and human eye. We and others have presented evidence that there are differences between types of mice in their susceptibility to RGC damage from elevated IOP. We selected the CD1 mice since they are confirmed to be more sensitive to RGC death in the model than C57Bl/6, thereby giving a greater opportunity to identify possible protective effects. Protective effects will best be confirmed, however, in different strains of mice, in rats, and in non-human primates to determine how robust they are. The doses of losartan and enalapril used here were selected due to past published work suggesting that they had measureable effects on connective tissue remodeling (in Marfan syndrome mice). Both lowered blood pressure quite significantly, and larger doses or longer treatment may have different effects, potentially causing hypotension. Higher systemic doses are limited by drug solubility and tolerability by the animals. Without further confirmation in clinical research, this study should not be interpreted to indicate that losartan or other sartans would be beneficial in patients with glaucoma, since there are surely differences by species in response to IOP. We are presently participating in large insurance database research to add data to this issue.

In summary, losartan substantially decreased RGC death in the mouse model of experimental glaucoma, despite lowering of vascular perfusion as calculated from higher IOP and lower blood pressure. The protective effect was associated with significant differences in the baseline state and remodeling of the sclera by losartan compared to control eyes. Losartan’s neuroprotection was associated with a decrease in axonal transport obstruction at the ONH, indicating that its mechanism includes reduced stress on RGC at that site, though an additional effect within the retina may be a contributing feature. Blockade of both AT1R and AT2R by enalapril led to lower blood pressure and greater RGC loss than control glaucoma eyes, suggesting that the beneficial effect of losartan was via a pathway involving inhibition of pERK.

## Supporting Information

S1 Fig Original western blots. (TIF)

S1 Table Raw data from mouse bead glaucoma drug treatment study. (XLSX)
