# Supplementary figures and images for: Losartan Treatment Protects Retinal Ganglion Cells and Alters Scleral Remodeling in Experimental Glaucoma
Source: PLoS One. 2015 Oct 27;10(10):e0141137. doi: 10.1371/journal.pone.0141137 (PMC4624713; doi:10.1371/journal.pone.0141137)

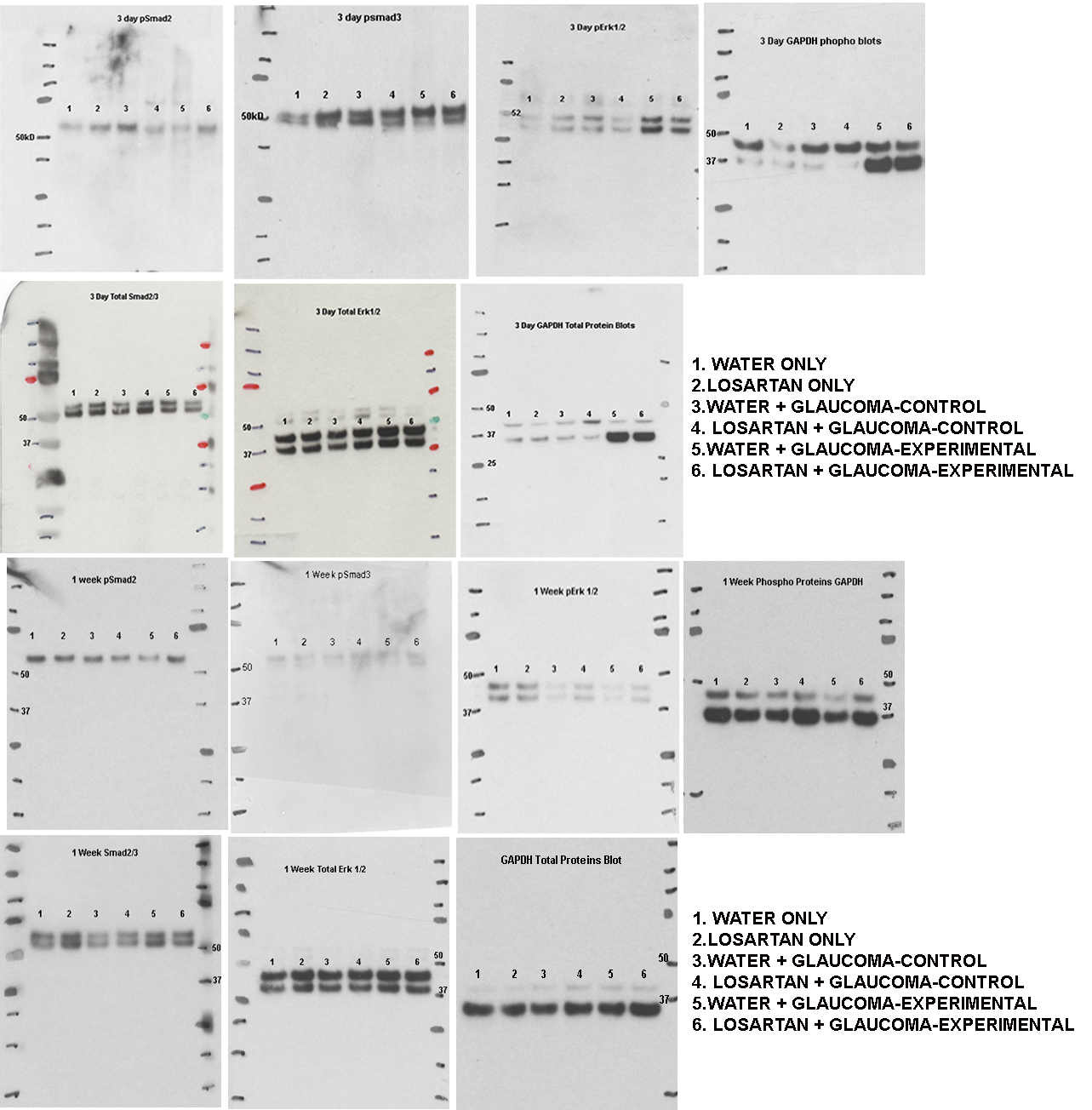

Supplement: S1 Fig — (TIF) [file pone.0141137.s001.tif]
